# The Missing Vein: A Case of Inferior Vena Cava Agenesis Presenting With Venous Thromboembolism

**DOI:** 10.7759/cureus.86897

**Published:** 2025-06-27

**Authors:** Antony Kimani, Wandera M Carey, Rajiv Patel, Soraiya Manji, Alok Iyer, Zamanali Khakhar, Sayed K Ali

**Affiliations:** 1 Department of Internal Medicine, Aga Khan University Hospital, Nairobi, KEN; 2 Department of Medicine, University of Nairobi, Nairobi, KEN

**Keywords:** agenesis of the ivc, azygos–hemiazygos, deep venous thrombosis (dvt), inferior vena cava (ivc), pulmonary embolism (pe), venous thromboembolism (vte)

## Abstract

Agenesis of the inferior vena cava (IVC) is a rare congenital anomaly that is frequently identified incidentally in patients presenting with venous thromboembolism. The IVC is susceptible to a wide range of congenital anomalies. Due to its rarity and infrequent clinical encounters, it is often overlooked. We describe the case of a young man of African descent who presented with a deep vein thrombosis due to a previously unrecognized diagnosis of IVC agenesis. This case sheds light on IVC agenesis as an alternative diagnosis, especially in younger patients without traditional risk factors for venous thromboembolism.

## Introduction

Thrombosis is a major cause of morbidity and mortality and underlies several conditions, most notably ischemic heart disease, ischemic stroke, and venous thromboembolism (VTE) [1]. VTE is a multifactorial condition resulting from a complex interaction of genetics and environmental exposures that trigger thrombosis with subsequent embolization. In Africa, the prevalence of VTE is estimated to be between 2.4% and 9.6% with traditional risk factors such as surgery and pregnancy responsible for most cases [2]. In Kenya, the mean age of VTE is estimated to be about 40.6 years, with a peak between 35 and 50 years [3], emphasizing that the incidence is even lower in those aged 20-35 years. When major risk factors for DVT are ruled out in this age group, genetic factors as well as congenital causes are sought. 

The inferior vena cava (IVC) is a large vein responsible for venous return predominantly from the lower extremities, abdominal viscera, and pelvic organs and is prone to a wide spectrum of congenital anomalies such as duplication, interruption, and absence, which can lead to VTEs [4]. IVC agenesis accounts for approximately 5% of idiopathic DVT in young patients without associated risk factors [5]. We present a case of a late presentation of DVT with an underlying etiology of an absent IVC. The overlooked diagnosis of IVC agenesis appears to have been a significant contributory factor in the development of deep venous thrombosis in this otherwise clinically unremarkable patient. Due to the uncommon nature of true caval agenesis, there is limited literature on this condition in Sub-Saharan Africa. With the intent of deepening the understanding of the clinical presentation and management of this seldom-reported vascular anomaly, this case aims to contribute meaningfully to the evolving discourse surrounding this underexplored condition. Traditionally, treatment approaches have primarily focused on medical management, including anticoagulation and compression therapy.

## Case presentation

A 29-year-old male of African descent, with no prior history of DVT, presented with a long-standing history of bilateral lower limb varicose veins, first noted in 2015, now extending to the groin and suprapubic regions. He previously used graduated compression stockings, which provided partial symptomatic relief. The patient denied dizziness or shortness of breath on exertion. However, he reported heaviness and progressive pain in the legs after prolonged walking, with progression of varicosities following physical activities such as mountain climbing or jogging. The patient had a body mass index (BMI) of 22.7 kg/m^2 ^and reported no history of smoking, alcohol consumption, or illicit drug use. The patient denied any family history of pulmonary embolism (PE) or DVT.

On examination, his vitals were stable. There was no pallor or lymphadenopathy on examination. His cardiovascular and respiratory examinations were unremarkable. Abdominal exam revealed no organomegaly and no pain on palpation. Musculoskeletal examination was significant for bilateral lower limb varicosities in both supine and erect positions, extending to the thighs and suprapubic region bilaterally. There was the presence of bilateral calf tenderness, with partially healed bilateral venous ulcerations at the medial malleoli. The patient underwent further diagnostic workup, including laboratory investigations, as summarized in Table 1.

**Table 1:** Patient laboratory results. MCV, mean corpuscular volume; MCH, mean corpuscular hemoglobin; MCHC, mean corpuscular hemoglobin concentration; RDW, red cell distribution width; PT, prothrombin time; INR, international normalized ratio; APTT, activated partial thromboplastin time; BUN, blood urea nitrogen

Test	Results	Reference
White blood count	5.65 × 10^9^/L	4.00-10.00 × 10^9^/L
Neutrophils (%)	57.3%	40.00-80.00%
Lymphocytes (%)	27.3%	20.00-40.00%
Monocytes (%)	8.3%	2.00-10.00%
Eosinophils (%)	6.2%	1.00-6.00%
Basophils (%)	0.9%	0.00-2.00%
Neutrophils absolute	3.24 × 10^9^/L	2.00-7.00 × 10^9^/L
Lymphocytes absolute	1.54 × 10^9^/L	1.00-3.00 × 10^9^/L
Monocytes absolute	0.47 × 10^9^/L	0.20-1.00 × 10^9^/L
Eosinophils absolute auto	0.35 × 10^9^/L	0.02-0.50 × 10^9^/L
Basophils absolute auto	0.05 × 10^9^/L	0.02-0.10 × 10^9^/L
Red blood count	4.51 × 10^12^/L	4.50-5.50 × 10^12^/L
Hemoglobin	13.7 g/dL	13.00-17.00 g/dL
Hematocrit	41.0%	40.00-50.00%
MCV	90.9 fL	83.00-101.0 fL
MCH	30.4 pg	27.00-32.00 pg
MCHC	33.4 g/dL	31.50-34.50 g/dL
RDW	12.7%	11.60-14.00%
Platelet Count	255.0	150.00-410.00 × 10^9^/L
PT	12.30 seconds	10.50-13.30 seconds
INR	1.04	<2.00
APTT	31.50 seconds	24.13-35.10 seconds
Thrombin time	15.9 seconds	14.6-19.0 seconds
Sodium	138.20 mmol/L	136.00-145.00 mmol/L
Potassium	4.52 mmol/L	3.50-5.10 mmol/L
Chloride	104.30 mmol/L	98.00-107.00 mmol/L
Bicarbonate	24.30 mmol/L	22.00-29.00 mmol/L
BUN	4.07 mmol/L	3.20-8.20 mmol/L
Creatinine	84.30 µmol/L	62.00-115.00 µmol/L

Although factor V Leiden, prothrombin gene mutation, antiphospholipid antibodies, and protein C and S deficiencies were considered in the diagnostic workup, testing was deferred due to significant financial constraints. Serologic testing revealed negative results for hepatitis B surface antigen, hepatitis C antibody, HIV-1 and HIV-2 antibodies, and antinuclear antibody.

**Figure 1:**
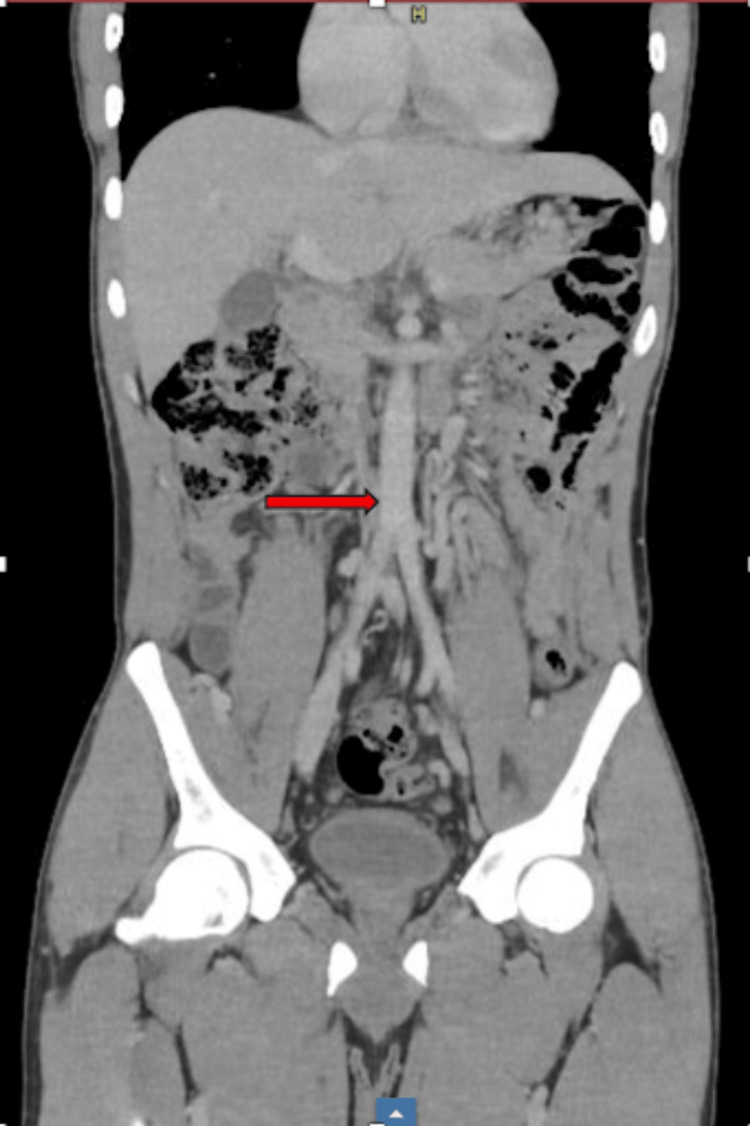
Coronal CT image of the abdomen and pelvis with contrast. The inferior vena cava (IVC) is not visualized along its expected anatomical course to the right of the aorta (red arrow).

**Figure 2:**
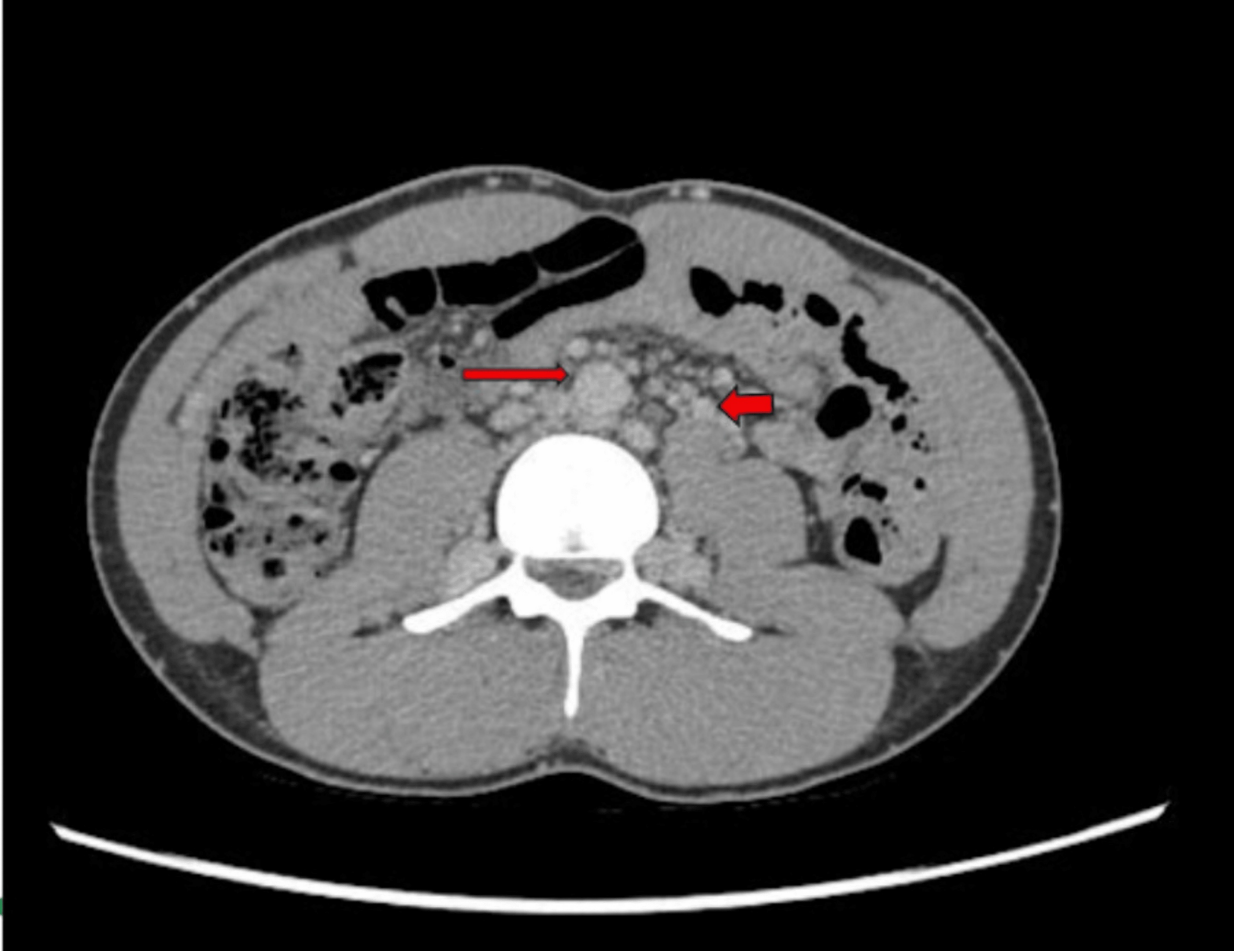
CT scan demonstrating absence of the IVC with extensive collateral venous development as a compensatory mechanism, consistent with IVC agenesis. IVC, inferior vena cava

A bilateral lower extremity Doppler ultrasound revealed features consistent with DVT involving both superficial and popliteal veins. These findings, in the absence of other underlying conditions, prompted further investigation for a possible underlying malignancy. An abdominal CT scan was performed, which demonstrated a dysplastic IVC (Figure 1). This was accompanied by extensive collateral venous drainage within the abdomen, pelvis, and abdominal wall (Figure 2). Prominent azygos and hemiazygos venous pathways were also noted. No abdominal or pelvic lymphadenopathy was observed. 

The patient was initiated on therapeutic-dose enoxaparin at 1 mg/kg twice daily and later discharged on rivaroxaban 15 mg twice daily. He was advised to avoid strenuous physical activity. Supportive measures, including the use of compression stockings, were recommended. At his two-week follow-up, he was clinically stable, with moderate improvement in lower extremity swelling and pain.

## Discussion

The formation of the IVC is a complex and highly coordinated process that occurs during early embryogenesis, involving sequential development, regression, and fusion of three paired venous structures. From eight weeks of gestation, these venous systems undergo selective regression, anastomosis, and remodeling to form the fully developed IVC, consisting of four primary segments, moving in a caudal direction: hepatic, suprarenal, renal, and infrarenal. Due to the complexities that take place during its formation, variations in its final structure can occur. Such anomalies are found in approximately 0.3% of healthy individuals and 0.6% to 2% of patients with other cardiovascular abnormalities [5,6]. As a compensatory mechanism, collateral venous pathways, particularly the azygos and hemiazygos systems, persist and enlarge, forming alternative routes for venous drainage, as observed in the current patient [7]. In some cases, these prominent collaterals can exert a mass effect, presenting clinically as low back pain [8].

The development of DVT in individuals with IVC agenesis is primarily attributed to venous hypertension and increased hydrostatic pressure, which contribute to chronic venous insufficiency and thrombus formation. Additional mechanisms include venous stasis resulting from reliance on slow, high-resistance collateral pathways, such as the azygos-hemiazygos system, leading to pooling of blood in the lower extremities. Mechanical compression by enlarged collaterals may also contribute, as these vessels can impinge on adjacent structures, including paravertebral veins, further exacerbating venous congestion. In some cases, this has been associated with radiologic findings of paravertebral or paratracheal masses [9]. 

Bilateral DVT is observed in approximately 50% of patients with IVC agenesis [10], underscoring the significant role of underlying anatomical abnormalities in thrombus formation. Pulmonary embolism is relatively uncommon in this population, as emboli are often trapped within the azygos-hemiazygos collateral system before reaching the pulmonary circulation. In many reported cases, a triggering factor for acute thrombosis is identified, with strenuous physical activity being the most common, occurring in up to 20.6% of cases [1]. During intense exertion, the collateral venous pathways may be unable to accommodate the increased blood flow demand, resulting in venous stasis and subsequent thrombus formation [11].

The diagnosis of congenital absence of the IVC (IVCA) is likely underrecognized, often due to incomplete radiologic evaluation. In patients presenting with DVT, IVCA cannot be identified using standard compression B-mode ultrasonography and requires further assessment with cross-sectional imaging, such as CT or MR venography. In one center in Italy, a diagnostic protocol was implemented in which all patients with thrombus extending above the femoral ligament on ultrasound underwent contrast-enhanced CT. This approach led to the diagnosis of IVCA in five patients [9]. This strategy, however, is based on the experience of a single institution. Consequently, there is a need for broader implementation studies to evaluate the diagnostic utility of contrast-enhanced CT in identifying IVC agenesis. CT imaging offers the added benefit of excluding pelvic or abdominal masses, making it the modality of choice in evaluating patients with DVT, particularly in bilateral cases, such as the one presented here.

Due to the rarity of IVCA, no standardized treatment protocol exists for associated DVT. Management primarily focuses on preventing thrombus formation, progression, and recurrence. Standard therapy typically involves low molecular weight heparin (LMWH) followed by oral anticoagulation, commonly vitamin K antagonists, combined with leg elevation and compression therapy. A case series from France reported symptomatic improvement in pain and edema among IVCA patients treated with prolonged vitamin K antagonists, with or without elastic stockings. Although not immediately life-threatening, lifetime anticoagulation is often recommended due to persistent discomfort and a lifelong risk of recurrent DVT, necessitating regular assessment for bleeding risk. Patient education on modifiable risk factors, such as prolonged immobilization and oral contraceptive use, is also important. Data on DVT recurrence rates are limited [11]. Alternative anticoagulants, including direct factor Xa inhibitors, have shown promise; for example, a case from Portugal reported no recurrence at three-year follow-up. In the current case, factor Xa inhibitor (rivaroxaban) was preferred over vitamin K antagonists and other anticoagulants due to its ease of use and superior patient compliance. The use of vitamin K antagonists necessitates regular and timely INR monitoring with subsequent dose adjustments. Rivaroxaban eliminates the need for frequent laboratory testing, thereby minimizing potential compliance issues and reducing the overall financial burden on the patient [12]. Additionally, catheter-directed thrombolysis (CDT) has been described as effective in reducing thrombus burden and providing rapid symptom relief compared to anticoagulation alone, with no major bleeding complications reported [13].

Currently, there are no established guidelines for its management. Treatment focuses on conservative management and prevention of thrombus formation and recurrence [14]. Regular follow-up facilitates early detection of DVT, enabling timely intervention and reducing the risk of further thrombotic events and ensuing complications.

## Conclusions

The occurrence of unexplained or bilateral DVT in young patients without identifiable comorbidities signals the importance of considering IVC agenesis as a potential underlying etiology. There exists a significant void in evidence-based guidelines to inform optimal anticoagulation duration, standardized treatment protocols, diagnostic approaches, and long-term management strategies for patients with IVC agenesis. Comprehensive clinical studies and further research are essential to deepen our understanding of this condition and guide the development of standardized therapeutic strategies aimed at improving long-term outcomes.
